# Increasing influenza vaccination coverage in healthcare workers: a review on campaign strategies and their effect

**DOI:** 10.1007/s15010-020-01555-9

**Published:** 2020-12-07

**Authors:** Sofie Schumacher, Jon Salmanton-García, Oliver A. Cornely, Sibylle C. Mellinghoff

**Affiliations:** 1 Faculty of Medicine and University Hospital Cologne, Department I of Internal Medicine, Excellence Center for Medical Mycology (ECMM), University of Cologne, Herderstraße 52-54, 50931 Cologne, Germany; 2 Faculty of Medicine and University Hospital Cologne, Cologne Excellence Cluster On Cellular Stress Responses in Aging-Associated Diseases (CECAD), Chair Translational Research, University of Cologne, Cologne, Germany; 3 German Centre for Infection Research (DZIF), Partner Site Bonn-Cologne, Cologne, Germany; 4 Faculty of Medicine and University Hospital Cologne, Clinical Trials Centre Cologne (ZKS Köln), University of Cologne, Cologne, Germany; 5 Center for Molecular Medicine Cologne (CMMC), University of Cologne, Cologne, Germany

**Keywords:** Healthcare personnel, Seasonal influenza, Influenza virus, Immunization, Vaccine uptake rate, Immunization programs

## Abstract

**Purpose:**

Increasing influenza vaccination coverage in healthcare workers is a challenge. Especially during the ongoing COVID-19 pandemic, high vaccination coverage should be attained. This review analyzed strategies to increase influenza vaccination coverage in healthcare workers.

**Methods:**

A literature search using PubMed was conducted and 32 publications on influenza vaccination campaigns for healthcare workers were reviewed for key interventions and resulting vaccination coverage.

**Results:**

Among key interventions analyzed, mandatory vaccination policies or multifaceted campaigns including a vaccinate-or-wear-a-mask policy as well as mandatory declination reached vaccination coverage in healthcare workers of over 90%. Although campaigns solely based on education and promotion or on-site-vaccination did not regularly exceed an absolute vaccination coverage of 40%, a substantial relative increase in vaccination coverage was reached by implementation of these strategies.

**Conclusion:**

Mandatory vaccination policies are effective measures to achieve high overall vaccination coverage. In clinics where policies are infeasible, multifaceted campaigns comprising on-site vaccination, vaccination stands and educational and promotional campaigns as well as incentives should be implemented. Lessons learned from influenza campaigns could be implemented in future SARS-CoV-2 vaccination campaigns.

## Introduction

Influenza is a highly contagious disease, causing 4.0–8.8 respiratory deaths per 100 000 individuals annually worldwide [1]. Vaccination is the most effective form of influenza prevention. Children under 5 years of age, chronically ill and immunocompromised patients, the elderly (> 65 years) and pregnant women are at high risk of complicated influenza courses. The World Health Organization (WHO) recommends annual influenza vaccination for these vulnerable populations as well as healthcare workers (HCW) [2]. HCW may transmit influenza to vulnerable patients, thereby compromising patient safety [3].

Despite this recommendation, vaccination rates among HCW are low ranging from 15.6 to 63.2% (median 30.2%) in Europe [4]. Other than allergies against vaccine compounds, there are no medical contraindications for influenza vaccination. If allergy to egg protein is known, a cell- or recombinant-based vaccine can be used [5]. The challenge is addressing personal reasons among unvaccinated staff against influenza vaccination. In the German OKaPII study, doctors stated mainly organizational aspects, whereas nurses declared lacking confidence in efficacy and safety of vaccines [6]. Organizational and educational issues can be approached and overcome. It should, therefore, be possible to increase influenza vaccination rates.

Most university hospitals in Germany treat high numbers of vulnerable patients. As immunocompromised patients may have an impaired immune response to vaccines, herd immunity is even more important [7]. To protect these patients, high influenza vaccination rates in HCW have to be achieved [8]. At the University Hospital of Cologne, we are planning an intensified influenza vaccination campaign for the upcoming season 2020/2021. Therefore, we analyzed the current literature on influenza vaccination campaigns for HCW.

In context of the ongoing coronavirus disease 2019 (COVID-19) pandemic, public health implications of the influenza season 2020/2021 must be considered. Coinfections of severe acute respiratory syndrome coronavirus 2 (SARS-CoV-2) and influenza virus have been described [9]. Sick leaves of HCW due to influenza or coinfections with SARS-CoV-2 could impact workforce availability. This, in combination with high infection rates in patients, could overburden our healthcare systems. Thus, high influenza vaccination rates among HCW should be attained [10].

## Methods

**Fig. 1:**
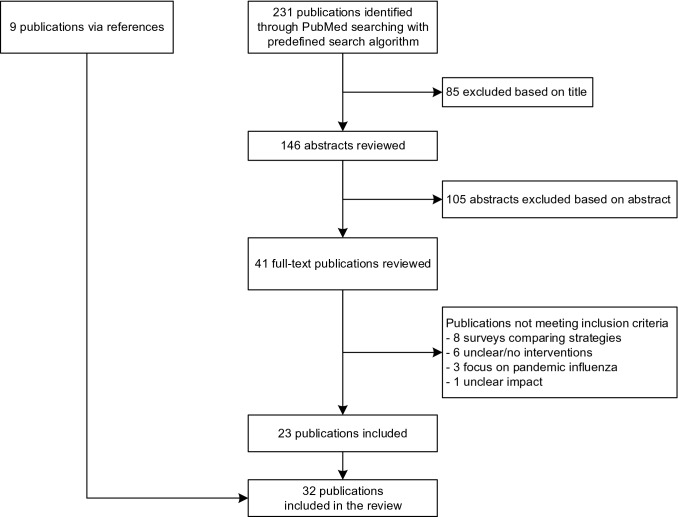
Study selection flow. Flowchart showing the study selection after searching with the following predefined search algorithm on PubMed®: [(“health personnel/analysis” (MeSH Terms) OR “health personnel/statistics and numerical data” (MeSH Terms)] AND “influenza, human/prevention and control” (MeSH Terms)] NOT [review (Publication Type)]. Additionally, nine publications were found through references of relevant publications

To identify influenza vaccination campaign strategies, we performed a literature search using the PubMed® database. The following query was defined: ((“health personnel/analysis” [MeSH Terms] OR “health personnel/statistics and numerical data” [MeSH Terms]) AND “influenza, human/prevention and control” [MeSH Terms]) NOT (review [Publication Type]). Articles published from January 2010 to August 2020 were included. No language restrictions were applied. Publications were selected by screening title and abstract. Studies implementing interventions to increase seasonal influenza vaccination rates among HCW were included. Studies focusing on pandemic influenza in 2009–2010 were excluded. The interventions needed to be clearly defined. Also, the selected studies had to include an evaluation of effect in comparison to a control group or in comparison to at least one previous season. Studies which conducted surveys in a number of institutions comparing different campaign strategies among each other were excluded. If the studies differentiated between nursing homes and acute care hospitals, we focused on acute care hospitals. Additionally, references of relevant publications were examined to identify further suitable studies (Fig. 1).

Each publication was reviewed for key interventions and resulting vaccination coverage (VC). VC was defined as the proportion of the vaccinated population in relation to the entire study population. The interventions of interest were education and promotion, incentives, organization, and policies. Education and promotion included providing material and spreading awareness. Incentives included free vaccine or giving away prizes among the vaccinated. Organizational interventions contained on-site vaccination, peer-to-peer vaccination, mass vaccination events and assignment of dedicated staff. Mandatory vaccination, vaccinate-or-wear-a-mask policies and declination forms to be submitted by unvaccinated HCW were grouped under policies. Moreover, combinations of these interventions were examined. The effect of the implemented strategy was evaluated by comparing VC before and after intervention. The relative increase in percent between initial and resulting VC was considered to evaluate the potential increase in VC regarding the key interventions.

## Results

### Literature search

Our initial search yielded 231 publications. After screening titles and abstracts, 41 publications remained. These full-text articles were assessed and subsequently 23 studies were included. Additionally, 9 publications found through references of relevant literature were added. In total, 32 articles were reviewed (Fig. 1).

Of the selected studies, 14 were conducted in the USA, 5 in Italy, 3 in Australia, and 1 each in Canada, France, Germany, Israel, Japan, Korea, Qatar, Spain, Switzerland, and Turkey. Most (*n* = 28) studies compared VC before and after a specific vaccination campaign conducted at individual or clustered institutions. Other studies (*n* = 4) compared vaccination rates between an intervention group and a control group. The majority (*n* = 30) of the studies were performed in hospitals, while two studies only analyzed nursing homes. In the following sections, the outcome per key interventions is described. Further details such as number of subjects described in each study can be found in Table 1. One study is listed under two key interventions [11].

**Table 1:** Summary of interventions, study population, season (year) and vaccination coverage

Publication	Country	Season	*N*	% VC			Relative increase		Key intervention	Key elements
				Before	After	Average ± SD [Range]	[%]	Average ± SD [Range] [%]		
Abramson et al. [12]*	Israel	2007–08	163	27	53	From 31.2 ± 26.3 [10–83] VC before, to 45.8 ± 27.3 [14–95] VC after	96.3	65.9 ± 55.8 [14.5–162.5]	Education/ Promotion	Lecture, reminders, literature via e-mail, personal interaction
Borgey et al. [13]*	France	2014–15	496	28	34		21.4			Slide shows and posters
Costantino et al. [14]*	Italy	2016–17	38	16	42		162.5			Training course
Cozza et al. [15]	Italy	2012–13	1813	10	14		40.0			Posters, fact sheets, online presence
Jung et al. [16]	Korea	2015–16	1433	83	95		14.5			Education, one-on-one counselling, on site vaccination
Llupia et al. [17]	Spain	2008–09	4500	23	37		60.9			Education, humorous pictures of vaccinated staff
Podczervisnki et al. [11]	United States	2011–12	1586	85	92	From 85 VC before, to 92 VC after	8.2	8.2	Incentive	Incentive
Barbara et al. [23]	Italy	2016–17	1013	10	18	From 11.0 ± 1.4 [10–13] VC Before, to 23.3 ± 11.2 [17–40] VC after	80.0	113.6 ± 102.7 [30.8–263.6]	Organization	On site vaccination
Gilardi et al. [27]	Italy	2017–18	2131	13	17		30.8			On site vaccination
Oguz et al. [25]	Turkey	2017–18	572	11	40		263.6			On site vaccination
Vimercati et al. [26]*	Italy	2017–18	700	10	18		80.0			On site vaccination
Awali et al. [31]	United States	2011–12	3054	80	93	From 74.0 ± 19.0 [40–99] VC before, to 87.8 ± 12.7 [58–99] VC after	16.3	25.0 ± 31.3 [31.1–97.9]	Policy	Mandatory vaccination
Babcock et al. [32]	United States	2008–09	25,980	71	98		38.0			Mandatory vaccination
Batabyal et al. [28]	United States	2008–09	29,000	48	66		37.5			Declination form
Batabyal et al. [28]	United States	2009–10	29,000	66	90	From 74.0 ± 19.0 [40–99] VC before, to 87.8 ± 12.7 [58–99] VC after	36.4	25.0 ± 31.3 [31.1–97.9]	Policy	Mandatory vaccination
Batabyal et al. [28]	United States	2010–11	29,000	90	62		− 31.1			Declination form
Batabyal et al. [28]	United States	2011–12	29,000	62	62		0.0			Declination form
Batabyal et al. [28]	United States	2012–13	29,000	62	86		38.7			Declination form
Batabyal et al. [28]	United States	2013–14	29,000	86	92		7.0			Declination form, vaccinate-or-mask
Batabyal et al. [28]	United States	2014–15	29,000	92	92		0.0			Declination form, vaccinate-or-mask
Batabyal et al. [28]	United States	2015–16	29,000	92	96		4.3			Declination form, vaccinate-or-mask
Esolen et al. [24]	United States	2009–10	12,363	47	90		91.5			Vaccinate-or-mask
Esolen et al. [24]	United States	2010–11	n.a	90	92		2.2			Vaccinate-or-mask
Esolen et al. [24]	United States	2011–12	n.a	92	95		3.3			Vaccinate-or-mask
Esolen et al. [24]	United States	2012-–13	19,985	95	97		2.1			Vaccinate-or-mask
Honda et al. [38]	Japan	2012–13	1616	87	97		11.5			Declination form
Huynh et al. [29]	United States	2010–11	5300	68	96		41.2			Mandatory vaccination
Kim et al. [34]	United States	2012–13	n.a	74	89		20.3			Declination form, vaccinate-or-mask
Ksienski [35]	Canada	2012–13	48,818	40	74		85.0			Vaccinate-or-mask
LaVela et al. [39]	United States	2013–14	173	54	77		42.6			Declination form
Modak et al. [36]	United States	2011–12	2723	61	85		39.3			Declination form, vaccinate-or-mask
Podczervisnki et al. [11]	United States	2012–13	1641	92	96	From 74.0 ± 19.0 [40–99] VC before, to 87.8 ± 12.7 [58–99] VC after	4.3	25.0 ± 31.3 [31.1–97.9]	Policy	Declination form
Quan et al. [22]	United States	2007–08	6414	44	63		43.2			Declination form, decentralized distribution, flu mobile
Quan et al. [22]	United States	2008–09	6734	63	58		− 7.9			Declination form, decentralized distribution, flu mobile
Quan et al. [22]	United States	2009–10	6568	58	87		50.0			Declination form, vaccinate-or-mask
Quan et al. [22]	United States	2010–11	6582	87	92		5.7			Declination form, vaccinate-or-mask, incentive
Rakita et al. [30]	United States	2005–06	5000	54	98		81.5			Mandatory vaccination
Rakita et al. [30]	United States	2006-–07	5000	98	99		1.0			Mandatory vaccination
Rakita et al. [30]	United States	2007–08	5000	99	99		0.0			Mandatory vaccination
Rakita et al. [30]	United States	2008–09	5000	99	99		0.0			Mandatory vaccination
Rakita et al. [30]	United States	2009–10	5000	99	99		0.0			Mandatory vaccination
Smith et al. [33]	United States	2011–12	30,000	71	97		36.6			Mandatory vaccination
Stuart et al. [37]	Australia	2013	208	47	93		97.9			Vaccinate-or-mask
Drees et al. [18]	United States	2011–12	10,286	66	92	From 67.0 ± 21.1 [17–94] VC before, to 71.4 ± 28.0 [17–94] VC after	39.4	14.4 ± 28.2 [-20–88.1]	Combined intervention	Declination form, vaccinate-or-mask, incentive, penalties, promotion, vaccination stations
Drees et al. [18]	United States	2012–13	10,388	92	94	From 67.0 ± 21.1 [17–94] VC before, to 71.4 ± 28.0 [17–94] VC after	2.2	14.4 ± 28.2 [− 20 to 88.1]	Combined intervention	Declination form, vaccinate-or-mask, incentive, penalties, promotion, vaccination stations
Drees et al. [18]	United States	2013–14	11,046	94	93		− 1.1			Declination form, vaccinate-or-mask, incentive, penalties, promotion, vaccination stations
Drees et al. [18]	United States	2014–15	10,883	93	93		0.0			Declination form, vaccinate-or-mask, incentive, penalties, promotion, vaccination stations
Friedl et al. [41]	Switzerland	2003–04	1322	n.a	20		n.a			Lectures, educational material, incentive
Friedl et al. [41]	Switzerland	2004–05	1163	20	17		− 15.0			Lectures, educational material, incentive
Friedl et al. [41]	Switzerland	2005–06	1236	17	30		76.5			Educational material, promotional material
Friedl et al. [41]	Switzerland	2006–07	1290	30	24		− 20.0			On site vaccination, lectures, educational material
Friedl et al. [41]	Switzerland	2007–08	1415	24	27		12.5			On site vaccination, educational material, incentive
Frisina et al. [42]	United States	2014–15	93	70	85		21.4			On site vaccination, educational material, Plan-do-study-act
Frisina et al. [42]	United States	2015–16	90	85	91		7.1			On site vaccination, educational material, Plan-do-study-act
Frisina et al. [42]	United States	2016–17	104	91	90	From 67.0 ± 21.1 [17–94] VC before, to 71.4 ± 28.0 [17–94] VC after	− 1.1	14.4 ± 28.2 [-20–88.1]	Combined intervention	On site vaccination, educational material, Plan-do-study-act
Frisina et al. [42]	United States	2017–18	102	90	90		0.0			On site vaccination, educational material, Plan-do-study-act
Gilardi et al. [27]	Qatar	2014–15	n.a	71	93		31.0			On site vaccination, mobile cart, education emails, group meetings, declination form
Heinrich-Morrison et al. [40]	Australia	2014	7480	56	80		42.9			Declination form, incentive, promotion, mass vaccination events
Horst-Schaper et al. [20]	Germany	2018–19	4000	n.a	n.a		n.a			Promotion, education, on site vaccination, incentive
Marshall et al. [21]	Australia	2013	8000	42	79		88.1			Declination form, incentive, “flu stop shop”, education
Marshall et al. [21]	Australia	2014	8000	79	80		1.3			Declination form, incentive, “flu stop shop”, education
Marshall et al. [21]	Australia	2015	8000	80	79		− 1.3			Declination form, incentive, “flu stop shop”, education
Marshall et al. [21]	Australia	2016	8000	79	80		1.3			Declination form, incentive, “flu stop shop”, education
Marshall et al. [21]	Australia	2017	8000	80	81		1.3			Declination form, incentive, “flu stop shop”, education
Marshall et al. [21]	Australia	2018	8000	81	82		1.2			Declination form, incentive, “flu stop shop”, education

Table of each reviewed publication with corresponding country, season, study population, VC before and after intervention and key intervention. The vaccination campaigns were evaluated regarding key elements and subsequently grouped under key interventions: education/promotion, combined interventions, incentive, policy, and organization. The average initial and resulting VC ± SD and range in regard to the key interventions is given. Additionally, the relative increase in percent between initial and resulting VC for each season is given. Also, the relative increase (with ± SD and range) in percent for each key intervention overall is given. Out of the 32 studies included, 4 studies were controlled interventional studies (indicated with *). For these studies, only the intervention arm was considered when comparing the VC.

*N* study population, *VC* vaccination coverage, n.a. not available, *SD* standard deviation

### Key intervention: education and promotion

Among the selected studies, six built their campaign mainly upon educational and promotional aspects [12–17]. Overall, the key intervention education and promotion increased VC relatively by 65.9% (standard deviation (SD): ± 55.8%, range: 14.5–162.5%) (Table1).

In one randomized trial from Israel, the intervention group (*n* = 163) received a lecture session, recurring emails containing literature as well as reminders and an appointed key figure from each department personally talked to each participant of the intervention group. Compared to the initial VC of 27%, the final VC was 53% in the intervention group. The VC in the control group increased from 20 to 27% [12]. A cluster-randomized controlled trial conducted in French nursing homes included slideshows and posters regarding prejudices against and reasons for influenza vaccination. VC increased from 28 to 34% in the intervention group. VC decreased from 24 to 23% in the control group [13]. A 1-h training course for all participants concerning influenza vaccination guidelines, vaccine types and administration was used as an intervention in an Italian study. Subsequently, the initial VC of 16% increased to a final VC of 42% in the intervention group. In comparison, VC increased from 13 to 31% in the control group [14]. A different Italian hospital appealed on personal as well as patient safety. It comprised posters in frequented areas, distribution of factsheets and intranet presence. Most survey participants (66%) agreed that the information was useful. Following the implementation of the toolkit, vaccination coverage was 14% which corresponded to earlier VC of 10% [15]. During a Korean campaign, unvaccinated HCW were contacted via phone for a ten-minute educational presentation. As this had no effect, unvaccinated medical doctors then received one-on-one educational counseling with on-site vaccination (OSV). VC increased from 83 to 93% [16]. In a Spanish before-and-after-trial, the key intervention consisted of a “I’ve already been vaccinated” webpage showing humorous pictures of all heads of departments as well as a vaccinated pregnant woman promoting vaccination also during pregnancy. The authors concluded that the campaign encouraged the discourse on vaccination increasing VC from 23 to 37% [17].

### Key intervention: incentives

Incentives were emphasized as a key intervention in one study. In six other studies, incentives were used as part of multifaceted campaigns [11, 17–22].

The above-mentioned study provided a 25 US Dollar gift card for every employee, if the overall VC reached 95%. This approach increased the VC from 87 to 92% [11]. The key intervention incentive increased VC relatively by 8.2% (Table1).

An employee-bonus program was implemented in two studies [18, 19]. A prize draw among vaccinated staff was part of two campaigns [17, 20]. In one multifaceted campaign, prizes where given to wards if the target VC was achieved [21]. One study took a different approach creating a disincentive for department leaders. Departments could lose budget allocations if vaccination rates were unsatisfactory. This increased vaccination rates from 87 to 92% [22].

The vaccine was offered free of charge in the respective prior season and during all included campaigns. Therefore, no aspect in this regard can be reported.

### Key intervention: organizational strategies

Organizational aspects which facilitated access to the vaccine were implemented in eight studies [18, 21–27]; however, OSV was highlighted as a main intervention in only four campaigns [23, 25–27]. OSV as implemented key intervention increased VC overall by 113.6% (SD: ± 102.7%, range 30.8%–263.6%) (Table1). An Italian teaching hospital introduced OSV observing an increase in vaccination rates in medical residents from 10 to 18% [23]. In a different Italian study, the VC increased from 10 to 18% in the intervention group after offering OSV. Of note, out of the vaccinated HCW, 80% received vaccination on-site. In comparison, VC increased by 1.5% in the control group (without offered OSV). Initial and resulting overall VC was not provided by the authors for the control arm [26]. At another Italian hospital, a promotional campaign as well as OSV had already been in place in previous seasons with VC of 13%. Increased availability of the vaccine through extended OSV as well as longer timeslots at vaccination stations and at the occupational health department were added increasing VC to 17% [27]. After offering OSV, the VC increased from 11 to 40% in a Turkish children’s Hospital [25].

The following studies used special organizational strategies as part of their campaigns and are discussed under their respective subheading. An approach using peer-to-peer vaccination was taken by two hospitals [22, 24]. A flu kit including the vaccine, consent forms and stickers was handed out to appointed team leaders of individual departments [24]. In the second clinic nurse managers could receive vaccines from the Occupational Health Department to distribute among their personnel [22]. A “flu-stop-shop” in a main area was organized in one Australian study. During the campaign, HCW could receive vaccination at the “flu-stop-shop” at all times without appointment [21]. In another study, a “blitz” campaign was conducted during the first 2 weeks of October. Vaccination stations were set up at all entrances of the hospital. Consequently, about 70% of all employees were vaccinated in the first 2 weeks [18].

### Key intervention: policies

Among the selected studies, 15 included policies as key interventions. Overall, policies increased VC relatively by 25.0% (SD: ± 31.3%, range 31.1%–97.9%) (Table1). One study conducted in the USA analyzed the effect of several different policies from 2008 to 2016. During seasons, in which policies included a signed declination option, the VC varied from 62 to 66%. Upon addition of educational aspects, VC increased to 86%. After a state-wide mandate in 2013, requiring unvaccinated staff to wear a mask, a maintained VC of 92–96% over the course of three seasons was reached [28].

In five studies, influenza vaccination was mandatory for HCW [29–33]. These publications were exclusively from the USA. Before implementation of the mandate, multifaceted vaccination campaigns had already been in place in all five studies with VC ranging from 54 to 80%. After influenza vaccination was made an employment requirement VC was 93–98%. In every study, “mandatory” implied that contracts with unvaccinated staff without exemptions were to be terminated. Overall, none to 0.14% of staff contracts were terminated due to the mandate. All five campaigns granted medical or religious exemptions. Egg allergy, history of Guillain–Barré syndrome and previously reported severe vaccine reaction were among the regarded exemptions. Exemptions due to medical reasons were acknowledged to 0.7–1.9% of staff and religious exemptions to 0.13–0.3%. One study declared that exemption requests reflected misinformation regarding the vaccine. These exemption requests included immunosuppression or pregnancy as reasons, although vaccination is recommended for both of these conditions [32]. Except for one hospital [29], the exempted unvaccinated staff had to wear a mask during influenza season.

A vaccinate-or-wear-a-mask approach was a key intervention in six publications [22, 24, 34–37]. A deadline for vaccination was set, after which unvaccinated staff had to wear a mask for the duration of the influenza season [34–36]. Vaccinated staff partially had markings on identification badges [22, 35, 36]. Supervisors were informed of their employees’ vaccination status and were held accountable in three campaigns [22, 24, 36]. One study implemented a 100 US Dollar fine for noncompliant staff [34]. Another study initially implemented contract termination as consequence of noncompliance, but was forced to retract due to litigation [35]. A sustained VC of 90–97% over 4 years, was achieved through a vaccinate-or-wear-a-mask policy in combination with a decentralized vaccine supply (complete vaccine kits for appointed team captains of different departments) in one study [24]. An Australian pilot study applied a vaccinate-or-wear-a-mask mandate in the nephrology department increasing VC from 47 to 93% (*n* = 208) [37]. Amid the six studies, three also included a declination form [22, 34, 36]. Overall, remarkable increases up to 97% in VC were observed after mask mandate [22, 24, 34–37].

Declination forms as a key intervention were used in three of the reviewed studies performed in Japan and the USA [11, 38, 39]. HCW refusing vaccination had to complete a declination form stating their reasons in all three studies. In a Japanese study noncompliant HCW, who neither received vaccine nor handed in declination forms, were interviewed by the hospital vice president. After implementing the mandatory declination form in this study, VC increased from 87 to 97% [38]. A pilot study conducted in a US Veterans Affairs facility included a signed statement acknowledging the personal risks and risks to others in their declination form. This study reported VC increasing from 54 to 77% [39]. Another study evaluated the impact of declination forms. Here, HCW refusing vaccination had to complete a 30-min educational module, receive one-on-one counseling and sign an attestation statement in presence of an occupational health or infection prevention staff. In cases of non-compliance, HCW were required to meet with their managers and a disciplinary letter was included in their employee file. This penalty-based approach increased VC from 92 to 96% [11]. Declination forms also played an important role in four multifaceted campaigns, which are discussed under the subheading “Combined interventions” [18, 19, 21, 40].

### Combined interventions

The following studies are campaigns which did not focus on one key intervention but rather implemented three or more interventions as multifaceted strategies (education/promotion, incentive, organization, and policies) [18–21, 40–42]. Overall, combined interventions increased VC relatively by 14.4% (SD: ± 28.2%; range: − 20 to 88.1%) (Table1).

For one campaign a task force led by the Infection Prevention Department incorporating Employee Health, Pharmacy, and Nursing departments among others was created. A new policy was implemented which required employees to fill out either a consent, declination or exemption form. This included attestation of vaccination elsewhere. Vaccinated employees were asked to wear a badge saying “I’m vaccinated because I care”. If the badge wasn’t worn, employees had to wear a mask, regardless of vaccination status. Noncompliance was considered in performance evaluations hindering possible promotions or raises. As a financial incentive, an employee bonus program was implemented. This multifaceted campaign increased VC from 57 to 72% (in the 3 years prior to the campaign) to 92–93% sustaining for four years [18].

Similarly, an Australian campaign consisted of multiple key interventions. For 6 months each year, a full-time influenza vaccination coordinator was employed. Appointed nurses conducted the vaccinations in aforementioned “flu-stop-shop”. An intranet page with educational and promotional input was created. Promotions were spread via intranet, stickers and posters across the hospital. The chief executive officer sent emails and held presentations promoting the campaign. If wards achieved target rates, they received prizes. A mandatory declination form was implemented. Managers had access to the vaccination status of their employees via a database and were expected to hold their employees accountable. During the 6 active years of this campaign, VC was 79% to 82% compared to a VC ranging from 42 to 48% before [21].

One hospital implemented a new multifaceted strategy on top of OSV, a mobile cart, educational input and recurring e-mails. They added educational group meetings and a mandatory declination form. Also, progress reports on VC were sent to managers and heads of departments informing them of unvaccinated staff, yet without consequences for noncompliance. This increased VC from 71 to 93% [19].

Another Australian study introduced a database to track vaccination status of all HCW, identification of unvaccinated staff on ID badges, a declination form and awards for VC margins reached in wards (coffee machines in case of more than 80% VC). Following this campaign, VC increased from 56 to 80% [40].

A German hospital initiated the “Be a flu fighter” campaign, thereby managing to increase their VC by 4.5-fold. Key interventions included promotion and education, mobile vaccination teams and prize drawings as incentives among the vaccinated staff. Through the implementation of the campaign, VC reached 72% in physicians and 50% in nurses. Baseline values were not reported [20].

One hospital in Switzerland reported their influenza vaccination campaign being unsuccessful. The campaign included: vaccination daily during lunchtime in the cafeteria for 2 weeks, individualized mobile vaccination appointments at wards or during meetings, a “health week”, incentives such as free lottery ticket or a free lunch, educational and promotional flyers and posters, influenza vaccine logo, intranet presence including “frequently asked questions”, involvement of the head nurse, personal letters to employees and recurring lectures. According to the authors, the multitude of interventions, however, did not significantly increase VC (increase from 20 to 27% over 5 years). Among nurses the VC even decreased due to fear of potential short- or long-term side effects and doubts of efficacy of the vaccine [41].

As part of a quality improvement study, several plan–do–study–act (PDSA) cycles over the course of four seasons were implemented in one US study. The campaign consisted of educational aspects such as the distribution of a fact sheet and personal discussions on vaccination with HCW. Second, vaccine availability was increased in general and specifically for night shift staff and staff in remote clinics. Also, communicational aspects were enforced by sending out monthly emails showing current influenza epidemiology with a reminder of the availability of vaccination. Because “fear of needles” was identified as a barrier during a PDSA cycle, nasal vaccination was provided reducing this obstacle. Overall, VC increased from 70% to over 90% [42].

### Descriptive comparison of key interventions

**Fig. 2:**
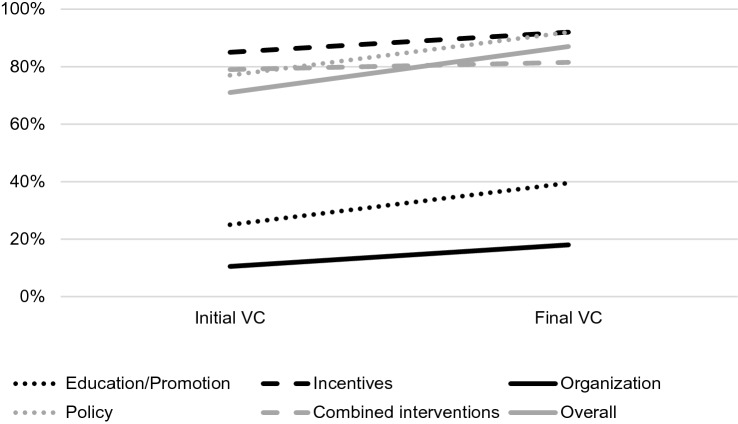
Median variation of vaccination rates after the application of different policies and overall. VC, vaccination coverage. Line graph of the initial and final VC in regard to implemented key interventions and overall. Education/promotion (*n* = 6) included providing material and spreading awareness. Incentives (*n* = 1) included prize draws. Organization (*n* = 4) included on-site vaccination. Policies (*n* = 15) included mandatory vaccination, declination form and vaccinate-or-wear-a-mask approaches. Combined interventions (*n* = 7) included combinations of the aforementioned interventions. Concerning the four controlled interventional studies, only the intervention arm was considered when comparing the VC in regard to the key intervention

As shown in Fig. 2, key interventions such as education or promotion (*n* = 6) and organization (*n* = 4) were used as interventions in campaigns with initially low VC (range 15–25%). Policies (*n* = 15) combined interventions (*n* = 7) and incentives (*n* = 1) were applied in studies with initially high VC (> 70%). In studies with low initial VC, the key intervention led to an increase of the VC ranging from 11 to 18% for organizational interventions and 25–40% for education/promotion. In studies with high initial VC, the key intervention led to an increase of the VC from 79 to 92% for policies and from 85 to 92% for incentives. No change was observed for combined interventions. In the overall group (*n* = 32, all studies), VC increased from 71 to 87%.

## Discussion

The analysis shows that vaccination campaigns are generally based on multifaceted vaccination strategies. Furthermore, vaccination strategies are implemented on different levels of initial vaccination rate. Most of the published vaccination strategies resulted in an increase in vaccination rates independent of the initial vaccination rate.

When taking into consideration, the overall success based on absolute VC, the most effective campaigns were those that comprised regulatory measures. Implementation of a mandatory vaccination policy generated the highest overall VC. Other policies like vaccinate-or-wear-a-mask or mandatory declination forms represented successful alternatives to mandatory vaccination. A VC of over 90% could be attained, especially if noncompliance with policies had a consequence [11]. Multifaceted campaigns which included a vaccinate-or-wear-a-mask approach as well as declination forms were generally more successful than those without policies. Policies were commonly used in Asia, Australia, and the USA.

Concerning the relative increase in VC regardless of initial and achieved VC, the most effective strategies were OSV (relative average increase 113.6%, SD: ± 102.7%, range 30.8–263.6%) and education/promotion (relative average increase 65.9%, SD: ± 55.8%, range 14.5–162.5%). However, regarding OSV, one outlier has to be considered: after implementation of OSV in a Turkish hospital, VC increased from 11 to 40% [25]. When comparing the potential relative increase between key interventions, the heterogeneity of the studies as well as the unbalanced number of studies in regard to key interventions has to be considered. Also, the studies with the key interventions OSV as well as education/promotion did not compare longitudinal data and mostly only focused on one season. In comparison, studies which employed polices (relative average increase 25.0%, SD: ± 31.3%, range 31.1–97.9%) and combined interventions (relative average increase 14.4%, SD: ± 28.2%; range – 20 to 88.1%) showed an initial high increase in VC and a maintained high VC over the course of following seasons [18, 21, 24, 30, 42]. Furthermore, before the implementation of policies, multifaceted campaigns comprising educational and promotional aspects as well as OSV had already been in place in the respective studies. This shows that education and promotion as well as OSV are valuable tools to increase VC and should be implemented whenever possible. However, in regard to absolute VC achieved, the key interventions education/promotion and OSV did not regularly exceed a VC of 40%.

Campaigns without regulatory measures focused on organizational, educational and promotional aspects. OSV was identified as an important tool to increase VC [25]. Mass vaccination events were successful [18]. A decentralized vaccine supply using peer-to-peer vaccination was also used [22, 24]. Regarding further organizational aspects, the importance of strong leadership and representation of clinic directors and heads of departments was stressed [21, 40]. Also, the importance of a dedicated team was highlighted in almost all studies. One study explicitly recommended hiring a physician solely dedicated to the influenza campaign over the course of the season [20]. Educational and promotional aspects were used as the basis of all campaigns, but when implemented as the sole key intervention absolute VC did not exceed 40% [17]. Incentives alone were rarely used as a key intervention, but did play an important role in multifaceted campaigns. Among the studies without policies, two studies stood out regarding overall VC achieved. One attained a VC of > 90% by implementing one-on-one counseling in combination with OSV [16]. The other study conducted several PDSA cycles analyzing and addressing barriers [42]. Of note, both studies were conducted in settings with high baseline VC in Korea and the USA (Table 1). These findings are in line with other studies [43–45].

Mandatory vaccination policies are confronted with opposition and even litigations. Any form of policy implies tracking the vaccination status of employees. This alone is a highly controversial topic considering data protection and staff autonomy. In most European countries, mandatory vaccination policies would be hard to implement. Possibly, a vaccinate-or-wear-a-mask policy could be installed in the future considering the current COVID-19 mask policy. HCW are now sensitized on the importance of wearing a mask. A mask not only acts as an incentive to receive vaccination but also reduces influenza transmission [37]. Surprisingly, in a Swiss study, HCW partially preferred wearing a mask over receiving the vaccine [46]. Of note, a vaccinate-or-wear-a-mask approach might not be feasible as an incentive to receive the vaccine in the season 2020/2021 due to already established mask mandates in the context of the COVID-19 pandemic. Ethical implications regarding incentives like prize draws for vaccinated staff should be considered. Alternatively, incentives in an educational context like quizzes could be a way to encourage discourse on the topic. Besides these ethical considerations, monetary and human resources need to be regarded. Further research on the economic impact of HCW influenza vaccination on work absenteeism as well as nosocomial influenza transmission is needed. Conclusive studies could help to integrate and justify policies regarding influenza vaccination for HCW.

With the possibility of a SARS-CoV-2 vaccine, similar issues and barriers regarding HCW vaccination could arise. Lessons learned from influenza campaigns could help to implement successful SARS-CoV-2 vaccination campaigns in the future.

This review has limitations. First, we only searched the PubMed® database. Second, due to the heterogeneity of the studies, we had to subjectively match campaigns to key interventions. Many interventions were part of multifaceted campaigns and not studied as an individual intervention. Therefore, it is difficult to finally assess the individual impact and contribution of the key interventions. Third, we did not perform statistical analysis, but rather focused on describing the strategies and the VC individual campaigns yielded. Fourth, the used key interventions were not balanced with regards to initial VC.

In conclusion, an influenza VC of over 90% in HCW can be reached by mandatory vaccination policies and through multifaceted campaigns which include a vaccinate-or-wear-a-mask-approach as well as mandatory declination policies. Policies, however, are often met by great opposition. In clinics where policies are infeasible, multifaceted campaigns comprised of extensive and individualized OSV and vaccination stands, a thorough educational and promotional campaign as well as incentives should be implemented to aim for an improved VC.

Overall, HCW influenza VC in Europe is far from satisfactory [4]. Although increasing the influenza VC in HCW remains a challenge, it is of utmost importance to protect our staff and our patients.
